# Supplementary material for: Dynamic Changes in the Endocannabinoid System during the Aging Process: Focus on the Middle-Age Crisis
Source: Int J Mol Sci. 2022 Sep 6;23(18):10254. doi: 10.3390/ijms231810254 (PMC9499672; doi:10.3390/ijms231810254)
Supplement: Supplementary file 1 [file ijms-23-10254-s001.zip › ijms-1863699-supplementary.pdf]

Supplementary Material

Supplementary Figure 1

A WT

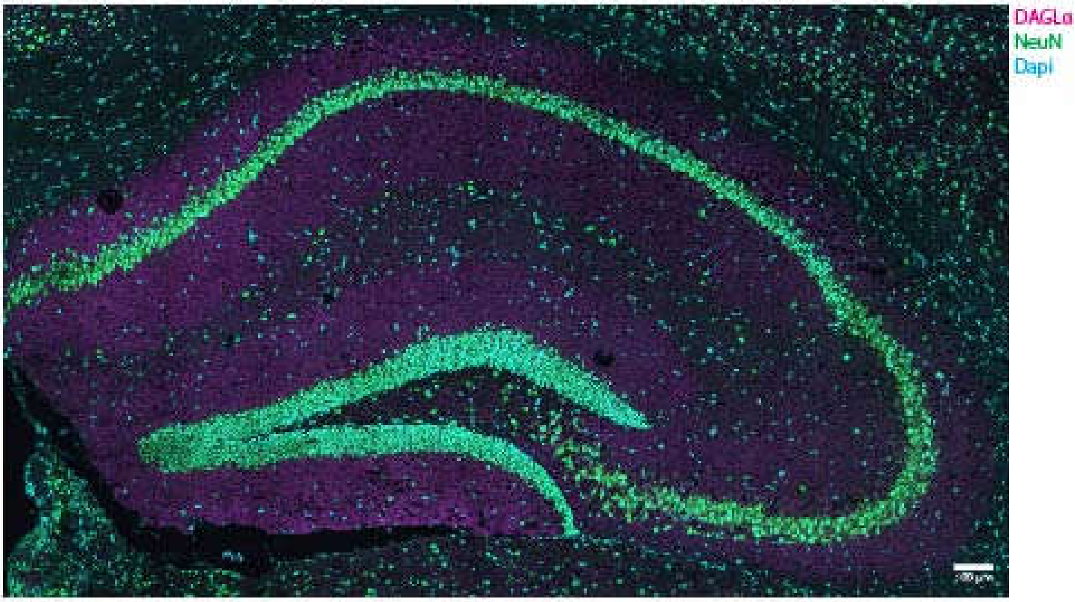

B DAGLα KO

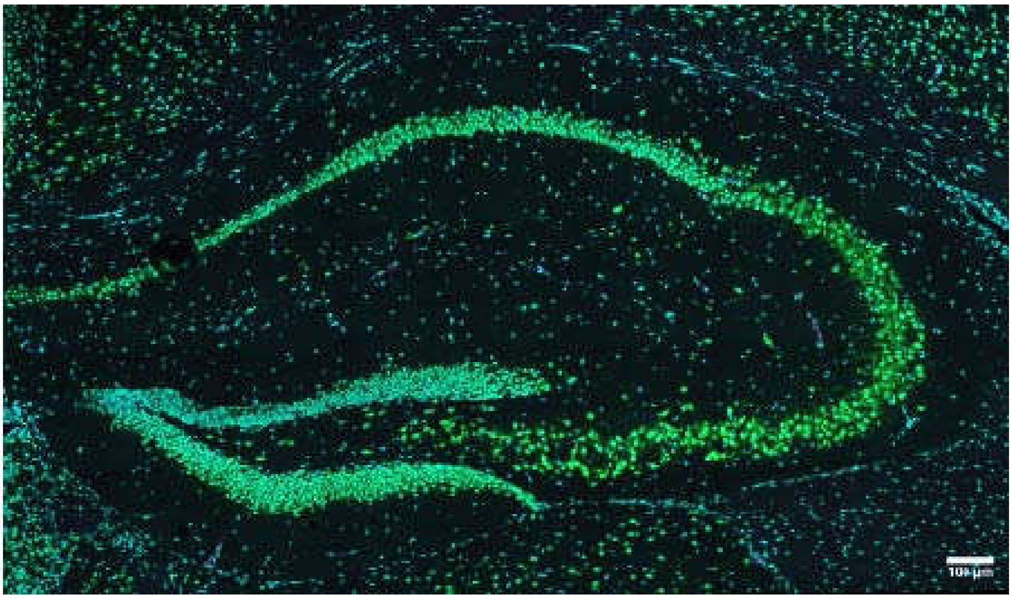

Supplementary Figure S1: Antibody control for DAGL∝. Coronal brain sections from WT and DAGL∝ KO mice immunostained for DAGL∝. Representative images depicting DAGL∝ specific immunoreactivity in the whole hippocampus (scale bar=100 µm) of the WT (**A**) and absence of DAGL∝ immunoreactivity in the KO mouse sections (**B**).

Supplementary Figure 2

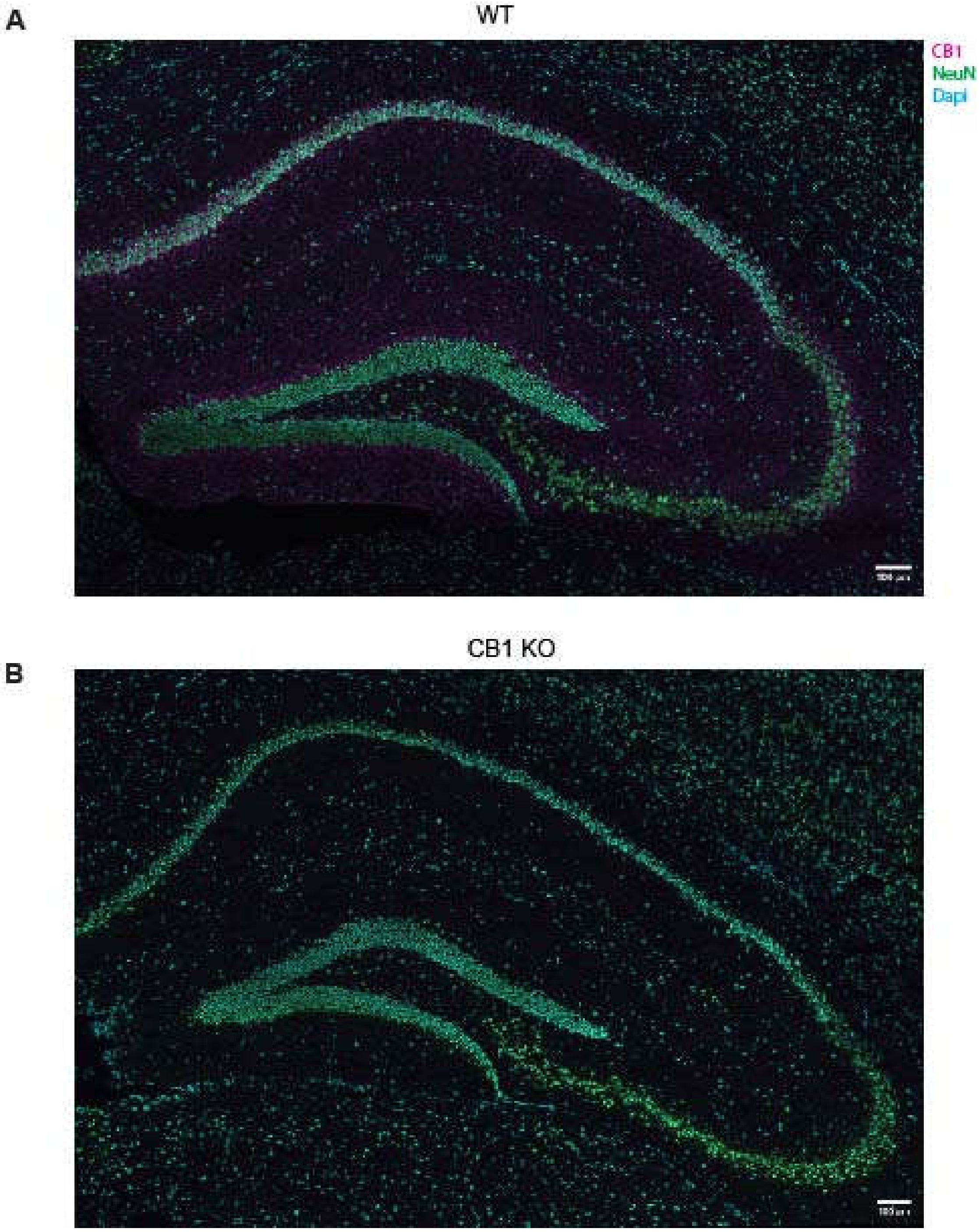

Supplementary Figure S2: Antibody control for CB1 receptor. Coronal brain sections from WT and CB1 KO mice immunostained for CB1 protein. Representative images depicting CB1 specific immunoreactivity in the whole hippocampus (scale bar=100 μm) of the WT (**A**) and absence of CB1 immunoreactivity in the KO mouse sections (**B**).
